# The assessment of data sources for influenza virologic surveillance in New York State

**DOI:** 10.1111/irv.12433

**Published:** 2016-11-14

**Authors:** Kay L. Escuyer, Christine L. Waters, Donna L. Gowie, Angie M. Maxted, Gregory M. Farrell, Meghan E. Fuschino, Kirsten St. George

**Affiliations:** ^1^ Laboratory of Viral DiseasesWadsworth CenterNew York State Department of HealthAlbanyNYUSA; ^2^ Bureau of Communicable Disease ControlNew York State Department of HealthAlbanyNYUSA

**Keywords:** computer models, data sources, influenza, surveillance

## Abstract

**Background:**

Following the 2013 USA release of the Influenza Virologic Surveillance Right Size Roadmap, the New York State Department of Health (NYSDOH) embarked on an evaluation of data sources for influenza virologic surveillance.

**Objective:**

To assess NYS data sources, additional to data generated by the state public health laboratory (PHL), which could enhance influenza surveillance at the state and national level.

**Methods:**

Potential sources of laboratory test data for influenza were analyzed for quantity and quality. Computer models, designed to assess sample sizes and the confidence of data for statistical representation of influenza activity, were used to compare PHL test data to results from clinical and commercial laboratories, reported between June 8, 2013 and May 31, 2014.

**Results:**

Sample sizes tested for influenza at the state PHL were sufficient for situational awareness surveillance with optimal confidence levels, only during peak weeks of the influenza season. Influenza data pooled from NYS PHLs and clinical laboratories generated optimal confidence levels for situational awareness throughout the influenza season. For novel influenza virus detection in NYS, combined real‐time (rt) RT‐PCR data from state and regional PHLs achieved ≥85% confidence during peak influenza activity, and ≥95% confidence for most of low season and all of off‐season.

**Conclusions:**

In NYS, combined data from clinical, commercial, and public health laboratories generated optimal influenza surveillance for situational awareness throughout the season. Statistical confidence for novel virus detection, which is reliant on only PHL data, was achieved for most of the year.

## 1 Introduction

Influenza surveillance is essential to monitor the spread and severity of the disease, identify populations at risk, detect the emergence of new subtypes and variant strains with pandemic potential, monitor the prevalence of drug resistance, characterize circulating virus types for the selection of strains for vaccine production, and provide information and guidance for clinicians and public health officials. The World Health Organization (WHO) established a global surveillance network for disease caused by influenza viruses in 1952.^1^ Since then, the Centers for Disease Control and Prevention (CDC) in collaboration with a network of PHLs has formed a US national influenza surveillance system comprising 85 PHLs performing molecular assays from the CDC to type and subtype the influenza virus, and 60 hospital laboratories reporting influenza test data.^2^ The consequent network and data monitoring systems have facilitated the detection of numerous important events and viral changes, including the rapid identification in 2009 of the pandemic influenza strain (A/H1pdm09). In 2010, to address concurrent fiscal constraints and emerging diseases, the CDC and the Association of Public Health Laboratories (APHL) initiated the Influenza Virologic Surveillance Right Size Project to assess the vast and complex national surveillance system, determine the most efficient means to monitor influenza activity, and establish a standard reference for the CDC and state PHLs.^2^ The Influenza Virologic Surveillance Right Size Roadmap (1st Edition released in 2013) attempted to guide surveillance toward a more systematic and statistically relevant process. The roadmap includes sample size calculators, developed to estimate the appropriate numbers of samples needed to achieve influenza surveillance with statistical confidence for situational awareness and rare/novel influenza event detection. The roadmap proposed identification of alternate, non‐PHL, data sources as a means to augment state PHL data and, in turn, enhance national surveillance.

Data generated from the NYS PHL, the Wadsworth Center, were measured against sample numbers calculated with the computer models for influenza situational awareness and rare/novel event detection. New York State alternate data from clinical and commercial laboratories were analyzed for integrity and impact on influenza situational awareness. Regional NYS PHL data were assessed for its impact on rare/novel event detection. New York State Department of Health scientific staff in the Virology Laboratory at the Wadsworth Center, in partnership with epidemiologists from the Bureau of Communicable Disease Control (BCDC), evaluated influenza testing practices, regulations, infrastructure, data collection, and reporting. Additionally, surveillance policy, potential future ideal practices and systems, and likely hurdles that may impede implementation were discussed.

## 2 Methods

### 2.1 Situational awareness of influenza viral disease in NYS

#### 2.1.1 Laboratory networks of influenza data sources

The NYS Wadsworth Center PHL performs influenza testing on specimens received through the Influenza‐like Illness Network (ILINet) and Emerging Infections Program (EIP), in addition to samples received from non‐EIP hospitals, student health clinics, veteran administration (VA) centers, long‐term care facilities, correctional facilities, and occasionally commercial laboratories.

**Figure 1:**
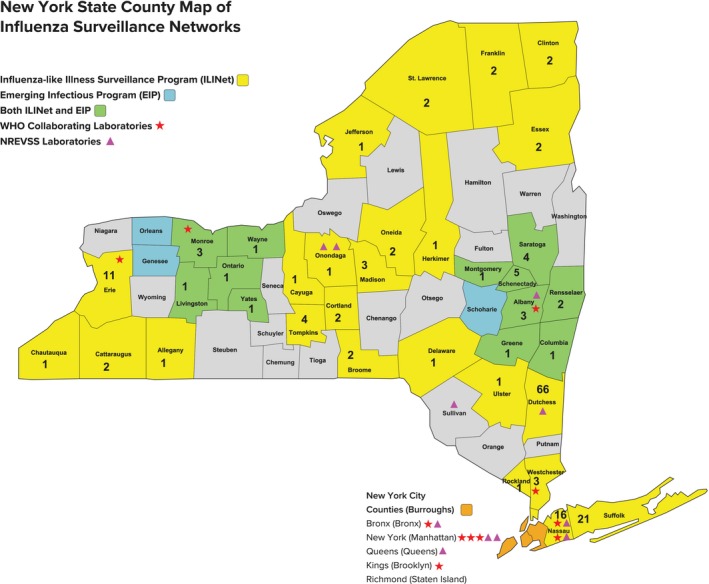
NYS map showing the 39 counties of 57 total outside of NYC that contribute to the ILINet and EIP influenza virologic surveillance networks. Counties participating in the EIP program are clustered around the cities of Albany and Rochester. The distribution of the ILINet primary care practitioners is indicated by number in each county and include the following practice types: pediatrics, family practice, internal medicine, student health, urgent care, obstetrics/gynecology, allergy and asthma, ear nose and throat, employee health, infectious disease, and pulmonology. Gray counties do not have providers enrolled in either the ILINet or EIP. The NYS map also depicts the 11 NREVSS laboratories and the 11 WHO collaborating laboratories, which include the NYS PHL in Albany and the three regional PHLs in Erie and Westchester Counties and NYC

The ILINet is an outpatient influenza surveillance program supported by CDC in all states.^3^ For the 2013‐2014 season, the NYSDOH ILINet Program had 173 participating primary care physicians (ILINet providers) in 39 of the 57 NYS counties outside of New York City, from a variety of medical practice specialties 4 (Figure 1). ILINet providers report data and submit specimens from patients with medically attended influenza‐like illness (MA‐ILI). The New York City Department of Health and Mental Hygiene (NYCDOHMH) coordinates a separate ILINet Program in the five counties of NYC.

The CDC supports the EIP, a program within FluServ‐NET,^3^ for surveillance of patients hospitalized with influenza. The influenza activities component of EIP in NYS comprises 21 hospitals in 15 counties around Albany in the Capital District region and Rochester in western NYS. Participating hospitals send a subset of data and influenza‐positive samples to the Wadsworth Center for confirmation and virus characterization.

The Wadsworth Center is both a WHO Collaborating Laboratory and a National Respiratory and Enteric Virus Surveillance System (NREVSS) laboratory. The 11 WHO and 11 NREVSS clinical laboratories in NYS (including four which are both) voluntarily participate in these networks and transmit influenza surveillance data to the CDC.^3^

#### 2.1.2 Sample size calculators

The Influenza Virologic Surveillance Right Size Project developed online Right Size Sample Size Calculators to determine optimal sample sizes, which should be analyzed to generate statistically meaningful, sufficient, and relevant data for influenza surveillance.^2^, ^5^ The user chooses the state or population, and based on the prevalence of influenza, the calculators generate the sample sizes with specified confidence levels, margin of errors (MOEs), and a reminder that biases in sampling could change the result. The CDC recognizes the start of influenza season at a threshold of ≥10% influenza positivity among all specimens tested from patients with MA‐ILI for two consecutive weeks. Calculator A utilizes this threshold to estimate the recommended sample sizes needed to ascertain influenza activity, with resulting information referred to as “situational awareness.” Calculator B determines statistically appropriate sample sizes for the detection of a rare/novel influenza virus.^2^

#### 2.1.3 Quality of influenza data

Considerable alternate data are readily available since NYSDOH added laboratory‐confirmed influenza to its list of reportable communicable diseases in December 2004. Positive influenza laboratory results, regardless of test method, are required to be reported to the NYSDOH 6 by clinical laboratories with permits. A NYSDOH clinical laboratory permit must be obtained through the Clinical Laboratory Evaluation Program (CLEP) by laboratories performing diagnostic testing on NYS patients,^7^ the requirements for which include a quality management system (QMS). NYS laboratories are eligible for (i) a permit for high or moderate complexity testing or (ii) registration as a Limited Service Laboratory (LSL) to perform waived tests that are simple and considered to have minimal risk of incorrect results or cause harm. Limited Service Laboratorys include nursing homes, school/student health services, dialysis facilities, ambulatory surgery centers, county health departments, correctional facilities, ambulance/rescue squads, and other direct patient care facilities. Physician Office Laboratories (POLs), operated by healthcare practitioners, only perform tests on specimens from their own patients and are exempt from the requirement to hold a CLEP permit. Limited Service Laboratorys and POLs, with minimal QMS and regulatory oversight, are not required to report positive influenza results to the state health department.

#### 2.1.4 Reporting of influenza data in NYS

The Electronic Clinical Laboratory Reporting System (ECLRS) provides an electronic system for prompt and protected transmission of reportable disease information to the NYSDOH, local health departments, and the NYCDOHMH.^8^

Clinical and commercial laboratories submit influenza‐positive test results electronically to the NYSDOH via ECLRS. Each ECLRS report contains specimen‐level data, including name, DOB, sex, address, home phone, county of residence, reporting laboratory, ordering physician, specimen source, testing method, results, specimen collection date, and report date. New York State Department of Health staff review the submitted data to determine whether it meets the case definition for laboratory‐confirmed influenza, defined as a positive influenza laboratory test result with at least one of the following methods: culture, enzyme immunoassay (EIA), direct immunofluorescence assay (DFA), immunofluorescence assay (IFA), RT‐PCR, immunohistochemistry (IHC), or influenza virus antigen detection systems (IVADs, also known as rapid influenza diagnostic tests, RIDTs). If the case definition is met, an influenza case report is created in the Communicable Disease Electronic Surveillance System (CDESS). The system automatically deletes duplicate CDESS case reports on the same patient. If the Wadsworth Center tests and does not confirm an initial positive influenza result from another laboratory, the initial test is considered a false‐positive result and the original influenza case report is revoked. Communicable Disease Electronic Surveillance System allocates ECLRS positive influenza laboratory results to disease classification codes for influenza type A or B, influenza type not specified, A/H1pdm09 subtype (since 2009), and H7N9 (since 2013). A disease classification code for influenza A/H3 subtype was added in October 2014 pursuant to discussions from this project.

While PHLs including Wadsworth use the CDC influenza rtRT‐PCR panel to detect and subtype influenza viruses, clinical laboratories use a variety of testing methods, which may or may not include subtyping. The majority of influenza molecular assays generate results in approximately 1‐8 hours and are capable of detecting influenza viruses with very high sensitivity and specificity; some tests also identify subtypes.^9^ Growth and isolation of influenza viruses in culture may take 7‐14 days or longer, while IVAD kits provide results in 15‐30 minutes.^10^ Table 1 summarizes 2014 information on testing platforms and assays, the number of licensed clinical laboratories using them in NYS, test complexity, and the influenza types/subtypes and other respiratory pathogens detected.

**Table 1:** Influenza testing platforms used by NYS CLEP licensed clinical laboratories testing NYS specimens

Influenza Testing Platforms																											
Data from NYS CLEP Proficiency Testing Event Jan 2014																											
	Molecular Nucleic Acid Detection										Culture								Antigen Detection								
	Real‐time RT‐PCR Typing/Subtyping Kits										Virus Growth, Isolation and IFA Confirmation								Influenza Virus Antigen Detection (IVAD)								
Number of labs performing each assay	50 labs total										32 labs total								208 labs total								
	16	8	6	3	3	1	9	1	1	2	2	14	2	4	3	3	1	3	53	50	30	30	29	5	5	4	2
Complexity of Assay High, Moderate, or Waived	Moderate	Moderate	High	High	High		Moderate	High	High	High	High	High	High	High	High	High	High	Waived	Waived	Moderate	Moderate/Waived	Moderate	Waived	Moderate	Waived	Waived	Waived
Name of Influenza Testing Assay, Respiratory Panel, or Kit	Cepheid Gene Xpert Flu	FilmArray (BioFire)	CDC Flu rRT‐PCR Dx Panel	GenProbe Prodesse ProFlu+^a^	GenProbe Prodesse ProFast+	Laboratory Developed^a^	Focus Simplexa Flu A/B RSV	Focus Simplexa Flu A H1N1	eSensor RVP (GenMark)	Luminex NxTAG^b^	D3 Duet DFA Inf. A/Respiratory Kit	D3 Ultra DFA Respiratory ID Kit	D3 DFA Influenza A Reagent	Diag. Hybrids/Quidel unspecified	Millipore/Light Diag. Inf. A&B DFA	Millipore Respiratory DFA Kit	Oxoid Imagen Influenza A/B Kit	Enzyme Immunoassay Kit^a^	Binax NOW Influenza A & B	BD Directigen EZ Flu A/B	BD Veritor System Flu A+B	Remel X/Pect Flu A & B	Quidel Quick Vue Influenza A & B	Meridian Bioscience TRU FLU	Quidel Sofia Influenza A+B	3‐M Rapid Detection/RAMP Flu	Quidel Quick Vue Influenza
**Virus Type/subtype Detected**																											
Influenza Type A	X	X	X	X	X		X	X	X	X	X	X	X		X	X	X		X	X	X	X	X	X	X	X	X
Influenza A/H1 seasonal		X	X		X				X	X																	
Influenza A/H3		X	X		X				X	X																	
Influenza A/H1pdm09	X	X	X		X	X		X	X	X																	
Influenza A/H5			X																								
Influenza A/H7			X																								
Influenza Type B	X	X	X	X			X		X	X		X			X	X	X		X	X	X	X	X	X	X	X	X
Adenovirus		X							X	X	X	X				X											
Human coronaviruses OC43, 229E, HKU1, NL63		X								X																	
Human metapneumovirus		X							X	X																	
Human rhino/enterovirus		X							X	X																	
Parainfluenza 1, 2, 3		X							X	X	X	X				X											
Parainfluenza 4		X								X																	
Respiratory syncytial virus (RSV)				X			X				X	X				X											
RSV Type A		X							X	X																	
RSV Type B		X							X	X																	

a In combination with other kits

b The Luminex xTAG, used in 2014, did not detect influenza A/H1pdm09, human coronaviruses or parainfluenza viruses.

### 2.2 Rare/novel influenza virus detection with NYS PHL data sources

Revised recommendations released in 2014 11 advised using only PHL data generated with molecular methods for the assessment of sample sizes for detection of a rare/novel influenza event. Three regional PHLs exist in NYS: the Erie County PHL in western NY, the NYC PHL, and Westchester PHL downstate. Sample sizes and confidence levels were evaluated with the calculators for all available data generated with the CDC influenza rtRT‐PCR assay from Wadsworth and the regional PHLs.

Pooling state surveillance data into national aggregates produce large enough sample sizes to meet recommended confidence levels for the detection of a rare/novel influenza event. During peak season when influenza positivity is 20% or greater, the Right Size Roadmap recommends that states calculate sample sizes sufficient to have 95% confidence in detecting one novel virus among 700 influenza‐positive specimens. Prior and post‐peak influenza activity, when positivity is less than 20%, the Roadmap recommends a detection threshold of one novel virus of 200 influenza‐positive specimens, while for off‐season and summer periods, a threshold of one novel virus among four influenza positive samples is recommended.

## 3 Results

### 3.1 Recommended sample sizes for NYS influenza surveillance

The goal for NYS is to obtain recommended sample sizes for the state population of approximately 20 million, which would ensure optimal detection thresholds with ≥95% confidence and ≤5% margin of error (MOE), for both situational awareness and rare/novel event detection (Table 2). Computer modeling software for situational awareness with Calculator A establishes ideal sample sizes using unscreened MA‐ILI specimens. For detection of a rare/novel event, the current Calculator B revised late 2015 uses only Flu+ specimens tested at state PHLs.

**Table 2:** Sample sizes calculated for NYS using the influenza Virologic Surveillance Right Size roadmap calculators A and B for 2013‐14

NYS sample sizes^a^Confidence level ≥95% and margin of error ≤5%	Full year	High season	Low season	Off‐season
		Detection thresholds		
		1/700	1/200	1/4
Estimated prevalence Flu+/MA‐ILI^b^ patients	10%	30%	10%	1%
Patient specimen type	MA‐ILI100%	Flu+100%	Flu+100%	Flu+100%
Percent of specimen type				
Calculator A: situational awareness for seasonal influenza				
Recommended sample size per week	137	‐	‐	‐
Weeks sample size attained by Wadsworth NY PHL	6	‐	‐	‐
Weeks sample size attained NY PHLs & WHO/NREVSS data	41	‐	‐	‐
Calculator B: detection of a rare/novel influenza event				
Recommended sample size per week	‐	130	37	1
Weeks sample size attained	‐	0	4	40^c^
Total weeks per season	‐	6	6	40

a All calculations based on population of 19 570 261.

b Flu+ = Laboratory confirmed influenza, MA‐ILI = medically attended influenza‐like illness.

c Off‐season 30 of 40 weeks if 1Flu+/week, or more accurately 40/40 weeks with 1 Flu+ every 5 weeks.

### 3.2 Sample size calculations for situational awareness in NYS

**Figure 2:**
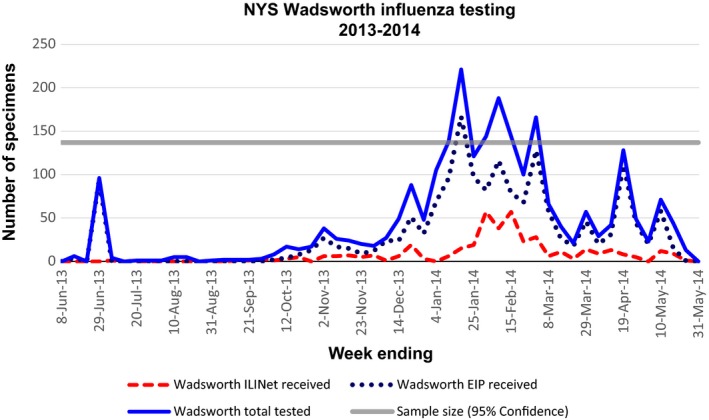
Influenza testing performed during 2013‐2014 by the Wadsworth Center on respiratory samples, relative to the recommended sample size determined from the Right Size Roadmap Calculator A for situational awareness, with 95% confidence and 10% expected prevalence of laboratory‐confirmed Flu+/MA‐ILI

New York State influenza test data were compared with the recommended sample sizes for situational awareness as determined with Calculator A (Figure 2). To avoid bias, specimens should preferably be unscreened, or a random sampling. The Wadsworth Center Virology Laboratory receives specimens for influenza testing from many sources including some that are prescreened by IVADs or other methods. During most weeks of peak influenza activity, sample sizes needed to achieve ≥95% confidence levels for situational awareness were obtained only with a combination of randomly submitted Flu+ and MA‐ILI specimens. During peak season, the recommended sample sizes were not achieved with only MA‐ILI specimens, or outside of peak season with Wadsworth test data alone.

**Figure 3:**
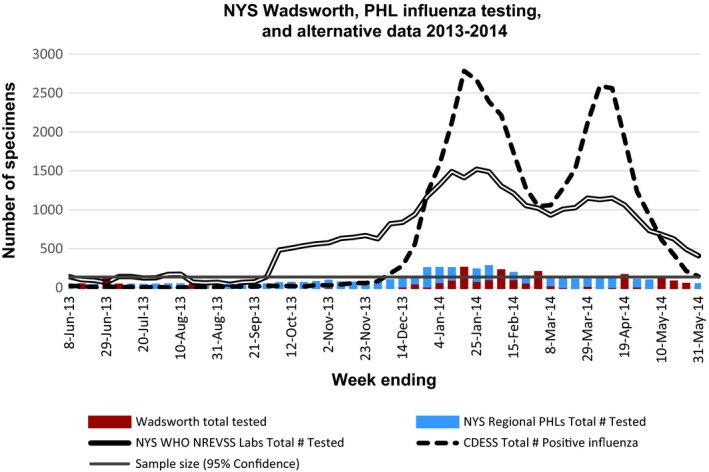
Influenza testing performed during 2013‐2014 by the Wadsworth Center, NYS regional PHLs, WHO/NREVSS‐collaborating laboratories in NYS, and clinical and commercial laboratories with NYS permits (CDESS data), relative to the recommended sample size determined with the Right Size Roadmap Calculator A for situational awareness, with 95% confidence and 10% expected prevalence of laboratory‐confirmed Flu+/MA‐ILI. (Sample numbers are reported by specimen received date for Wadsworth, collection date for WHO/NREVSS, and report date for CDESS)

The WHO/NREVSS laboratories in NYS provide additional data with sufficient power to meet the optimal confidence levels and MOE determined with Calculator A (Figure 3) during high and low influenza activity. While the WHO/NREVSS laboratories already transmit data to the CDC, the data from the WHO/NREVSS laboratories within NYS can still be used for state surveillance. In utilizing the WHO/NREVSS data to determine the true prevalence of Flu+/MA‐ILI, confidence levels were 99% ± ≤5% throughout the year, including off‐season when adjusted for estimated prevalence, indicating that the WHO/NREVSS data are likely to provide an accurate representation of the true influenza prevalence.

Alternate data for situational awareness during the 2013‐2014 influenza season in NYS were obtained from 193 clinical laboratories, which reported 43 281 positive influenza laboratory results to ECLRS, including results of high‐ and moderate‐complexity testing as well as waived testing. These results generated 37 180 positive influenza CDESS cases (Figure 3), which do not include negative influenza test results, yet are comprised of IVAD, culture, and molecular influenza testing methodologies. In NYS, a biphasic influenza picture occurred during the 2013‐2014 season with highest levels of influenza type A circulating in January and high levels of influenza type B circulating in April.

**Figure 4:**
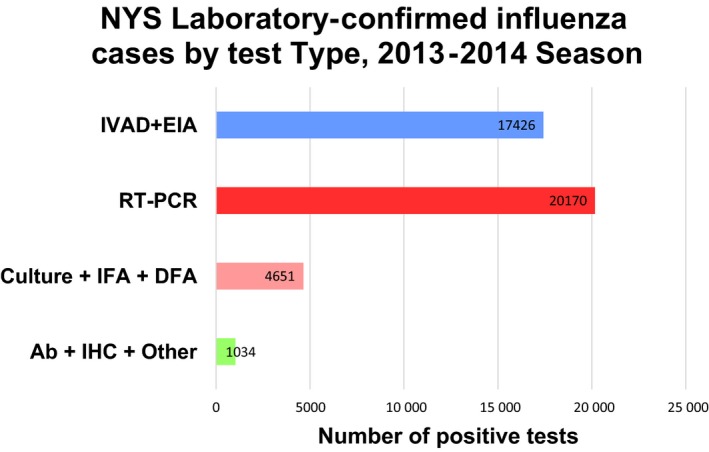
For the 2013‐2014 influenza season 43 281 total influenza positive tests were reported to ECLRS, of which 40% were positive by IVAD and EIA, 47% positive by PCR, 11% positive by culture, IFA, and DFA, and 2% by antibody, IHC, and other tests

Test methods for the 2013‐2014 season included 17 426 influenza test results positive by IVAD methods (40%) compared to 20 170 positive by PCR tests (47%) (Figure 4). The majority of clinical laboratories use IVAD tests for influenza. The impact of alternate data on situational awareness was analyzed for both rapid influenza tests and molecular test methods. Surpassing the situational awareness threshold of 137 samples to indicate the start of the influenza season, IVAD testing yielded 155 positive samples during the first week of December [Morbidity and Mortality Weekly Report (MMWR) week 1349], and 219 PCR‐positive samples 2 weeks later (MMWR week 1351). However, false‐positive IVAD results are of particular concern outside of peak influenza season when the positive predictive value of these tests is low.

### 3.3 Sample size calculations for rare/novel virus detection with NYS PHL data

**Figure 5:**
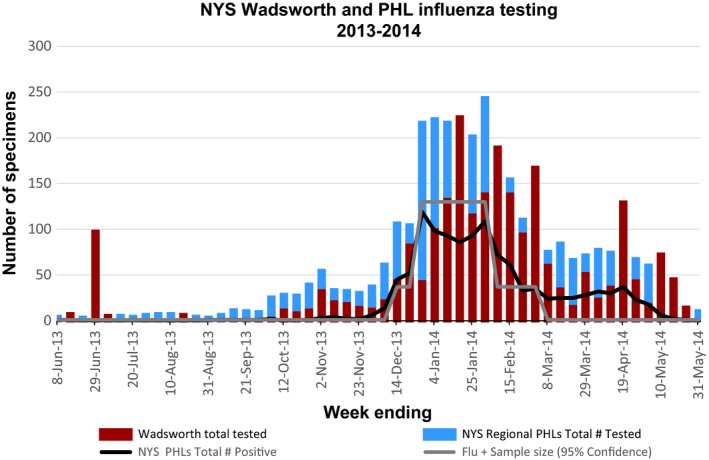
Influenza testing performed during 2013‐2014 by the Wadsworth Center, and NYS regional PHLs. Total number of positives are shown relative to the recommended Flu+ sample sizes determined from the Right Size Roadmap Calculator B for detection of a rare/novel event with 95% confidence and expected prevalence of 30% for high season (1/700 detection threshold), 10% for low season (1/200 detection threshold), and 1% for off‐season (1/4 detection threshold). (The elevated specimen numbers for Wadsworth for weeks ending June 29, April 19, and May 10, are due to one EIP submitter sending backlogged specimens)

For the detection of a rare/novel influenza virus, Wadsworth test data were compared to recommended sample sizes for NYS, aggregated on a national scale, determined from Calculator B (Figure 5). The total number of Flu+ specimens tested at the Wadsworth Center was insufficient to detect a rare/novel event for influenza surveillance at the recommended confidence levels. To augment detection of a rare/novel influenza virus, NYS regional PHL influenza rtRT‐PCR data supplemented the Wadsworth Center rtRT‐PCR data (Figure 5). From the last week of December 2013 through January 2014 with peak influenza activity, Flu+ data provided 86% to 94% confidence in the likelihood of detecting a novel virus present at 1/700 of cases. During low season, the recommended threshold for detection of a novel virus outside of peak season is 1/200 with a minimum Flu+ sample size of 37; sample sizes with 95% confidence were obtained for 4 of those 6 weeks (Table 2). During off‐season, the recommended threshold drops to ¼ with a minimum Flu+ sample size of 1. In fact, just 1 Flu+ sample is needed every 5 weeks per state, as 11 Flu+ samples are needed per week for 52 states nationally (personal communication, Lynette Brammer, CDC). Sample sizes with 95% confidence were obtained for 40 of 40 off‐season weeks when counting 1 Flu+ every 5 weeks. Thus, to contribute to national surveillance in detecting a rare/novel influenza virus, combined sample sizes from Wadsworth and the regional PHLs were sufficient to reach minimum confidence levels (≥85%) for recommended detection thresholds during peak weeks of influenza activity, and optimal (≥95%) confidence for the majority of low season and all of off‐season, yet not throughout the year.

## 4 Discussion

For patient samples tested at Wadsworth, the recommended sample size for influenza surveillance for situational awareness was achieved for half of the peak weeks of influenza activity. This data included test results from the random submission of specimens from hospitalized cases as well as those from primary care patients. To augment the total surveillance information, other data sources from clinical and commercial laboratories were investigated. The WHO/NREVSS‐collaborating laboratories, geographically spread throughout NYS, generate considerable data for situational awareness and directly transmit these data to the CDC, as well as NYS. All WHO/NREVSS laboratories post negative as well as positive results, providing sufficiently robust data for estimates of influenza prevalence. Beyond the WHO/NREVSS‐collaborating laboratories, the remainder of the clinical laboratories that report to ECLRS do not report to CDC, nor are their data shared with CDC by the NYSDOH. These ECLRS/CDESS data consist of only influenza‐positive cases, and sufficient numbers are not attained for situational awareness in the off‐season months. The CDESS Flu+ cases for NYS surveillance comprise a large alternate data source that is not currently transmitted to the CDC and provides an indicator of prevailing influenza strains.

In the months preceding peak influenza activity, more samples were tested by IVAD than PCR, yet the reliability of the IVAD data is questionable. Some providers submit IVAD‐positive specimens to the Wadsworth Center for PCR confirmation and identification of the subtype, particularly at the beginning of the season. A significant number of IVAD‐positive tests are not submitted for confirmation by PCR testing but are still reported as CDESS‐positive cases. The Right Size Roadmap companion document released in October 2014 “Using Alternative Data for Influenza Virologic Surveillance” states “Alternative sources should ONLY be used for determining situational awareness. Only PHL rRT‐PCR test data should be used to meet national novel influenza event detection thresholds”.^11^

Wadsworth molecular data were insufficient to achieve recommended confidence levels and thresholds for detection of a rare/novel event throughout 2013‐2014. Only the CDC RT‐PCR panel used at the state PHLs detects all influenza subtypes commonly circulating in humans, as well as A/H5 and A/H7 strains with kits provided for reflex testing. Therefore, only the CDC assays are likely to reveal an emerging novel virus. Wadsworth and the regional NYS PHL data combined met the Flu+ sample sizes needed to detect a novel virus at a 1/700 threshold with minimal (≥85%) confidence, for the five peak weeks of influenza activity. Desired sample sizes, thresholds, and optimal (≥95%) confidence levels were obtained for the majority of low season (1/200 threshold) and all of off‐season (¼ threshold). To increase off‐season influenza testing, NYSDOH issues an annual notice to NYS clinical laboratories, requesting submission of all clinical samples positive for influenza by any detection method during the summer to the Wadsworth Virology Laboratory for testing with CDC influenza rtRT‐PCR assay.

Extensive validation studies have shown that molecular detection by rtRT‐PCR is highly sensitive and specific for the detection and subtyping of influenza viruses.^12^ Large respiratory viral panel (RVP) assays have become increasingly popular for the testing of respiratory samples, despite a potential decrease in sensitivity compared to single‐target PCR assays. In a comparison of 11 898 respiratory samples tested by the CDC influenza rtRT‐PCR assay and the Luminex xTAG RVP, influenza A positive samples with low viral load were often not detected by the RVP and were mostly detected only by the CDC rtRT‐PCR.^13^ Relying on RVP data alone could therefore introduce inaccurate positivity rates. Although sensitivity and specificities vary between different RVPs such as the BioFire Film Array, GenMark eSensor, Luminex xTAG, and the new NxTAG, a significant advantage is the detection of multiple respiratory pathogens (Table 1).^14^

Compared to molecular and culture assays, the ease and rapidity of point‐of‐care antigen screening tests are perceived as beneficial for clinical management, particularly during periods of high prevalence.^10^ IVAD tests detect only the influenza virus type and do not distinguish the subtypes of influenza, nor would they identify a rare/novel emerging virus. In a meta‐analysis of 17 studies comparing A/H1pdm09 detection by IVAD to that with rtRT‐PCR, the estimated overall sensitivity of IVADs was 51% (ranging from 11% to 88%) (95% confidence interval) and the specificity was 98% (95% confidence interval).^15^ Another meta‐analysis of 159 similar studies assessing IVAD performance determined assay sensitivities to average 62.3% (95% confidence interval) and the specificity to be 98.2% (97.5%‐98.7% confidence interval.) Result accuracy was variable, depending on whether the specimen was collected from a child or an adult, as well as virus type and subtype.^16^ Thus, IVADs cannot reliably detect emerging influenza subtypes, and their widespread use presents a risk of missing the spread of a potentially pandemic strain. For influenza surveillance, recommended practices include the confirmation of IVAD results using molecular assays or cell culture at the beginning and end of the influenza season.

The NYSDOH has enhanced its statewide influenza surveillance by making laboratory‐confirmed influenza reportable and developing an electronic reporting system. Electronic Clinical Laboratory Reporting System provides a mechanism for timely reporting, improves completeness and accuracy of reports, and facilitates the identification of emergent public health problems. Limiting the reportability of laboratory‐confirmed influenza to clinical laboratories has ensured that the submitted test data have been performed under extensive QMSs, and provides tens of thousands of reports per season for extensive temporal and geographic coverage. During influenza season, the NYSDOH BCDC Influenza Surveillance Coordinator compiles a detailed weekly influenza report, which is posted online. This report includes geographic and demographic distribution of influenza cases; weekly and seasonal comparison of case numbers; testing and subtyping data from the WHO/NREVSS‐collaborating laboratories in NYS; Wadsworth Center antiviral resistance data; numbers of healthcare facility‐associated outbreaks; severity of disease; and any pediatric influenza‐associated fatalities.^17^

Challenges exist in communicating the reporting requirements to clinical laboratories testing NYS patients. The multiple laboratory classifications (permitted, LSL, POL) and different reporting requirements across diseases can create confusion. During peak influenza season, NYSDOH Statistical Unit staff must prioritize influenza data processing over other reportable diseases. Means of reporting of demographic data, LOINC^®^ and SNOMED^®^ coding, and test descriptions need to be standardized. The addition of codes for new strains, as they arise, improves the granularity and accuracy of reporting. Further, ECLRS reporting does not include the total number of specimens tested for denominator data. No other reportable communicable disease in NYS requires laboratories to report denominator data, which would be burdensome and might require a legislated regulatory change.

Multiple networks in NYS contribute to influenza surveillance on the state level, but only some of those networks transmit data to the CDC for national surveillance. Wadsworth Center data together with NYS alternate data sources portray a clear picture of influenza in the community and give confidence to the surveillance of influenza viruses circulating at any time in each county or region. The NYS alternate data could enhance national influenza surveillance for situational awareness due to its volume, geographic representation within NYS, and reliability. During 2013‐2014, NYS experienced a second wave of influenza activity, mostly type B, as intense and long lasting as the first wave of influenza A/H1pdm09 activity. Several other northeast states also experienced this second wave of activity, but most of the United States did not. NYS was the only state in the United States to report widespread activity to CDC for 24 consecutive weeks. Thus, the impact of the NYS alternate data is widespread, contributing to national and subsequently international influenza surveillance.
